# P-535. Dengue Fever in Children at a General Hospital from Buenos Aires, Argentina, During 2023- 2024 Dengue Season

**DOI:** 10.1093/ofid/ofaf695.750

**Published:** 2026-01-11

**Authors:** Martin Brizuela, Débora Montiel Ríos, Cora Deandreis, Carolina Salvo, Sandra Barreiro

**Affiliations:** Hospital General de Agudos Velez Sarsfield, Buenos Aires, Ciudad Autonoma de Buenos Aires, Argentina; Hospital General de Agudos Vélez Sarsfield, Buenos Aires, Ciudad Autonoma de Buenos Aires, Argentina; Hospital General de Agudos Vélez Sarsfield, Buenos Aires, Ciudad Autonoma de Buenos Aires, Argentina; Hospital General de Agudos Vélez Sarsfield, Buenos Aires, Ciudad Autonoma de Buenos Aires, Argentina; Hospital General de Agudos Velez Sarsfield, Buenos Aires, Ciudad Autonoma de Buenos Aires, Argentina

## Abstract

**Background:**

Dengue fever, caused by dengue virus (DENV 1-4) and transmitted by *Aedes aegypti* and *albopictus* mosquitoes, has become a persistent public health concern in Argentina. The aim of this study was to present the clinical and laboratory characteristics of dengue cases in patients under 15 years of age at a general hospital in Buenos Aires, Argentina during the 2023-2024 dengue season (EW 31/2023 and WE 30/2024, between July 30^th^ 2023 and July 27^th^ 2024).

**Methods:**

We conducted an observational, descriptive, and retrospective cohort study including patients under 15 years with laboratory-confirmed dengue diagnosis during the 2023-2024 dengue season. Statistical analysis was performed using RStudio.

**Results:**

This study included 243 patients. 60% (n=147) male, with a median age of 11 years old. 56% (n=136) of cases occurred between EW 12 and 15 (March 17^th^ to April 13^th^, 2024) and 97.5% (n=237) were managed and followed-up as outpatients. Positive diagnostic tests were NS1 antigen (n=207), IgM (n=39), and PCR (n=25). Clinical manifestations were 94% (n=228) mild, 6% (n=14) moderate, and 0.5% (n=1) severe. Myalgia was more frequent among children >6 years of age and rash among the group of 6 to 12 years of age (p< 0.05). Laboratory findings showed a median WBC count of 3,410/mm^3^, hematocrit 38%, and platelet count 168,000/mm^3^. No patients died, and one patient required transfer to another hospital due to alarm signs. Among the 6 hospitalized patients, the median age was 10 years (IQR 9- 12 years), and 85% (n=5) were males. The median hospital stay was 4.5 days. 70% (n=4) of patients were classified as moderate dengue with alarm signs.

**Conclusion:**

This retrospective study reveals the age-dependent clinical characteristics of dengue infection among pediatric patients. Our findings demonstrate that while dengue predominantly presents as a mild illness across age groups, there are significant variations in symptom presentation and immunological response. It is important to consider the difference according to the age of the patients. These insights can inform clinical strategies for pediatric dengue management in endemic regions, highlighting the need for tailored diagnostic and treatment approaches across different childhood age groups.

**Disclosures:**

All Authors: No reported disclosures

